# Initial Cluster of Novel Coronavirus (2019-nCoV) Infections in Wuhan, China Is Consistent with Substantial Human-to-Human Transmission

**DOI:** 10.3390/jcm9020488

**Published:** 2020-02-11

**Authors:** Hiroshi Nishiura, Natalie M. Linton, Andrei R. Akhmetzhanov

**Affiliations:** 1 Graduate School of Medicine, Hokkaido University, Kita 15 Jo Nishi 7 Chome, Kita-ku, Sapporo-shi, Hokkaido 060-8638, Japan; nlinton@gmail.com (N.M.L.); akhmetzhanov@med.hokudai.ac.jp (A.R.A.); 2 Core Research for Evolutionary Science and Technology, Japan Science and Technology Agency, Honcho 4-1-8, Kawaguchi, Saitama 332-0012, Japan

**Keywords:** epidemiology, transmissibility, zoonosis, cluster, exposure, statistical inference

## Abstract

Reanalysis of the epidemic curve from the initial cluster of cases with novel coronavirus (2019-nCoV) in December 2019 indicates substantial human-to-human transmission. It is possible that the common exposure history at a seafood market in Wuhan originated from the human-to-human transmission events within the market, and the early, strong emphasis that market exposure indicated animal-to-human transmission was potentially the result of observer bias. To support the hypothesis of zoonotic origin of 2019-nCoV stemming from the Huanan seafood market, the index case should have had exposure history related to the market and the virus should have been identified from animals sold at the market. As these requirements remain unmet, zoonotic spillover at the market must not be overemphasized.

## 1. Introduction

The clinical summary of the earliest cases of 2019 novel coronavirus (2019-nCoV) infections in Wuhan, China was recently published [1], showing the majority of cases were exposed to the Huanan seafood market, which also had wild animals, suggesting the possibility of zoonotic transmission in the market. This suggestion of zoonotic spillover was quoted by international organizations, including the World Health Organization (WHO), and as a result early research focused on zoonotic rather than direct human-to-human transmission of 2019-nCoV. However, the index case had no exposure history related to the seafood market, indicating that Huanan seafood market-related zoonotic spillover may have been an overblown hypothesis. Here, we reanalyze the epidemic data of the initial cluster of cases with 2019-nCoV infections to demonstrate that the epidemic curve is consistent with substantial human-to-human transmission in December 2019.

## 2. Epidemiological Analysis

**Figure 1:**
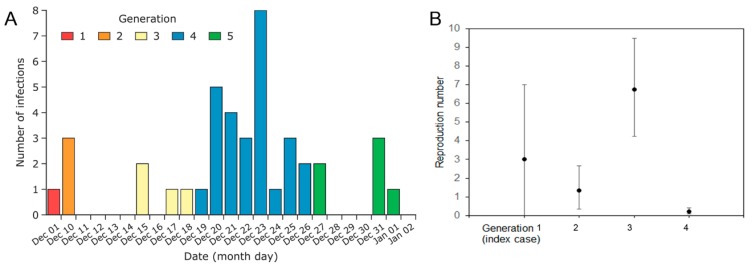
**The epidemic curve and estimated reproduction number by generation.** Generation 1 represents the index case. (**A**) The epidemic curve by date of illness onset [1]. A constant 8 days, counted from 10 December 2019, was used to define the generation-dependent number of cases. (**B**) The expected number of cases in each subsequent generation was assumed to follow a Poisson distribution, and the 95% confidence intervals of the reproduction number (whiskers) were derived from the profile likelihood.

Three important arguments are made here with respect to epidemiological interpretation of the epidemic dataset. First, Figure 1A shows the epidemic curve of cases in Wuhan, distinguishing case generations by color. The index case developed symptoms on 1 December 2019, with cases 2–4 having onset nine days later, and cases 5–6 five days after that. Together, these intervals indicate a possible serial interval (SI)—the time between illness onset in an earlier case to that in a secondary case—with a mean of 7.4 days, consistent with the mean SI of severe acute respiratory syndrome [2]. The latter is also consistent with the mean SI estimate of 7.5 days presented in the preliminary epidemiological study [3]. Although it is possible that the SIs are shorter than quoted here [4], the epidemic curve is still in agreement with the existence of asymptomatic and unascertained mild cases between diagnosed cases.

Second, assuming a constant SI of 8 days, the epidemic curve of cases by the date of illness onset can be transformed to that by generation of cases. The number of cases in each generation is therefore 1, 3, 4, 27, and 6 cases, respectively. These numbers allow for the estimation of generation-dependent reproduction numbers—the average number of secondary cases per primary case for each generation [5] (Figure 1B). Assuming that the offspring distribution is Poisson distributed, the reproduction numbers can be estimated at 3.0 (95% confidence interval (CI): 0.75, 7.8), 1.3 (95% CI: 0.4, 3.1), 6.7 (95% CI: 4.5, 9.6), and 0.2 (95% CI: 0.1, 0.5)—broadly in line with preliminary basic reproduction number estimates of 1.5–3.5 quoted by the WHO and presented elsewhere [6,7]. 

Third, the common exposure supports secondary transmission events taking place in the market. Although the virus has been identified in market environmental samples [8], this does not exclude the likelihood of secondary transmission. That is, it is possible that the common exposure history at the Huanan seafood market in Wuhan originated from the human-to-human transmission events within the market. 

## 3. The Take-Home Message for Outbreak Investigation

Unfortunately, early emphasis that market exposure implied animal-to-human transmission considerably delayed global recognition of exportation of the virus from Wuhan, especially during the first half of January [9,10]. The emphasis on market-based zoonotic transmission may have been the result of observer bias—i.e., the bias that originates from having preconceptions or subjective feelings about what is being studied that could influence epidemiological observation and even recording information. For example, the zoonotic origin of another relatively recently emerged coronavirus with predominantly zoonotic transmission—the virus causing Middle East respiratory syndrome (MERS)—may have served as a strong reference for reducing concern about epidemic levels of sustained human-to-human transmission.

In conclusion, we believe that zoonotic spillover at the market should not be overemphasized, because the epidemic curve is consistent with substantial human-to-human transmission in December 2019. There are two important take homes for any future investigations that begin with a similar scenario: first, to verify that zoonotic spillover is related to the exposure in question, the index case must be verified to have that exposure history. Second, without identifying the virus in animals sold at the market, it is difficult to conclude with certainty that any zoonotic transmission occurred at the market.
